# Direct Insertion of a Dumon Stent Into an Intermediate Bronchus Fistula From the Surgical Field

**DOI:** 10.1016/j.atssr.2024.04.001

**Published:** 2024-04-18

**Authors:** Ryo Sezaki, Takehisa Hashimoto, Masanori Tsuchida

**Affiliations:** 1 Niigata University Community Medicine Education Center, Uonuma Kikan Hospital, Niigata, Japan; 2 Division of Thoracic and Cardiovascular Surgery, Niigata University Graduate School of Medical and Dental Sciences, Niigata, Japan

## Abstract

A 54-year-old man underwent right S6 segmentectomy for right lung cancer. After discharge, he presented with fever, hemoptysis, and cough, and computed tomography showed an intermediate bronchus fistula. Because direct closure or bronchoplasty was challenging, a Dumon (Novatech) stent was inserted directly into the fistula from the surgical field and covered with an autologous pericardial patch, pedicled mediastinal fat, and intercostal muscle. The Dumon stent was removed by rigid bronchoscopy 1 year later. For an intermediate bronchus fistula that was difficult to repair by bronchoplasty, a Dumon stent was effective for maintaining bronchial patency and preserving the peripheral lung.

There have been few reports of postoperative bronchopleural fistulas other than bronchial stump fistulas, but they are reported to occur due to thermal damage from energy devices. In this case, an intermediate bronchus fistula after right S6 segmentectomy was treated with Dumon (Novatech) stent insertion from the surgical field and suture of an autologous pericardial patch.

A 54-year-old man was found to have a nodular shadow on chest radiograph during a routine health examination. Computed tomography (CT) showed a part-solid ground-glass nodule measuring 26x20 mm^2^ overall, with a high-density region of 13x10 mm^2^. There was no evidence of lymph node metastasis or distant metastasis. Endobronchial ultrasonography was inconclusive, and cytology showed class II findings. Because of clinical suspicion of primary lung cancer, video-assisted thoracoscopic right S6 segmentectomy with ND2a-1 was performed. The chest tube was removed on the second postoperative day, and the patient was discharged on the fourth postoperative day. The final pathologic diagnosis was minimally invasive adenocarcinoma, and the pathological stage was pT1aN0M0. The patient was readmitted with fever, hemoptysis, and cough, found to have a leukocytosis of 11,600, and CT findings concerning for a bronchial fistula (Figure 1A), which prompted bronchoscopy confirming the diagnosis (Figure 1B). On the 10th day after readmission, blood tests showed improvement, so it was decided to perform either temporary fistula closure or fenestration due to the extent of contamination in the pleural cavity. Reoperation was performed on the 13th day after readmission. Thoracotomy was performed through a posterolateral incision, and a large fistula extending from the membranous part of the intermediate bronchus to the right lateral wall was found (Figure 2A). Both the middle lobe bronchus and the basal bronchus opened just distal to the fistula. Because contamination of the thoracic cavity was mild, temporary fistula closure was performed. However, bronchoplasty was deemed difficult due to the proximity of the pulmonary artery. Therefore, a decision was made to insert a Dumon stent into the bronchus to maintain bronchial patency, and self-filling was necessary. A pericardial patch and mediastinal fat tissue were harvested, and 4-0 polydioxanone sutures were placed to avoid the pulmonary artery. The Y-type stent legs were cut to fit the lateral wall for the middle lobe and to be oblique for the basal segment (Figure 2B). The Dumon stent was inserted manually from the surgical field without using a rigid bronchoscope, ensuring that the stent's bifurcation touched the spur between the middle and lower lobe bronchi (Figure 2C). A thin bronchoscope (Olympus BF-290) capable of passing through a double-lumen tube was used to confirm that the legs of the stent were engaged in the middle and lower lobe bronchi. While the pericardial patch was sutured, the area close to the pulmonary artery side could not be completely sealed, so additional sutures were placed over the mediastinal fat tissue and the intercostal muscle of the seventh rib. After confirming by bronchoscopy that the middle lobe and basilar bronchi were open, and that there was no air leak, the dorsalis muscles were guided into the pleural cavity, and the operation was completed. The operative time was 6 hours 44 minutes, and blood loss was 1050 mL.

**Figure 1:**
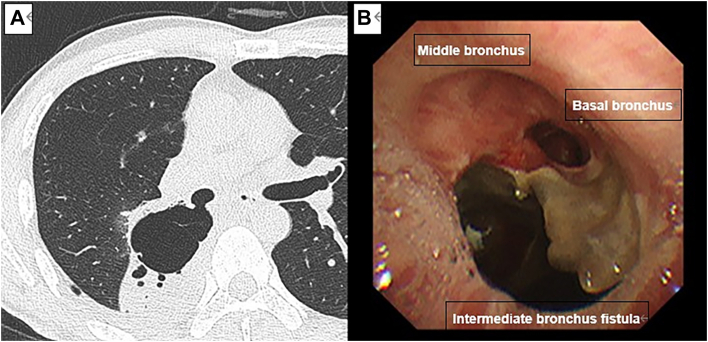
(A) Computed tomography and (B) bronchoscopy show the intermediate bronchus fistula.

**Figure 2:**
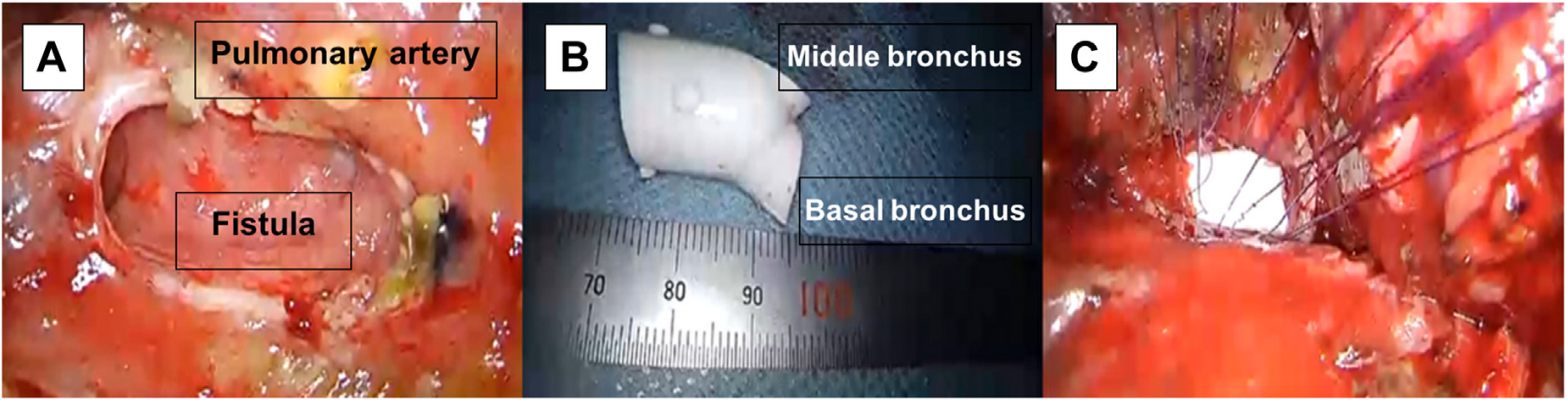
Surgical findings. (A) The intermediate bronchus fistula and adjacent pulmonary artery. (B) The cut Dumon (Novatech) stent. (C) The intermediate bronchus fistula with the cut Dumon stent inserted.

Because CT on the sixth day after the reoperation and bronchoscopy on the seventh day showed no problems with the stent’s position or lumen, the chest tube was removed. The patient was discharged on the 10th day after reoperation. On bronchoscopy 6 months after the reoperation and CT 10 months after the reoperation, no issues were observed with the stent’s position or lumen. Approximately 1 year after the reoperation, the stent was removed by rigid bronchoscopy (Figure 3). Subsequent follow-up showed no recurrence, and the patient is alive without recurrence.

**Figure 3:**
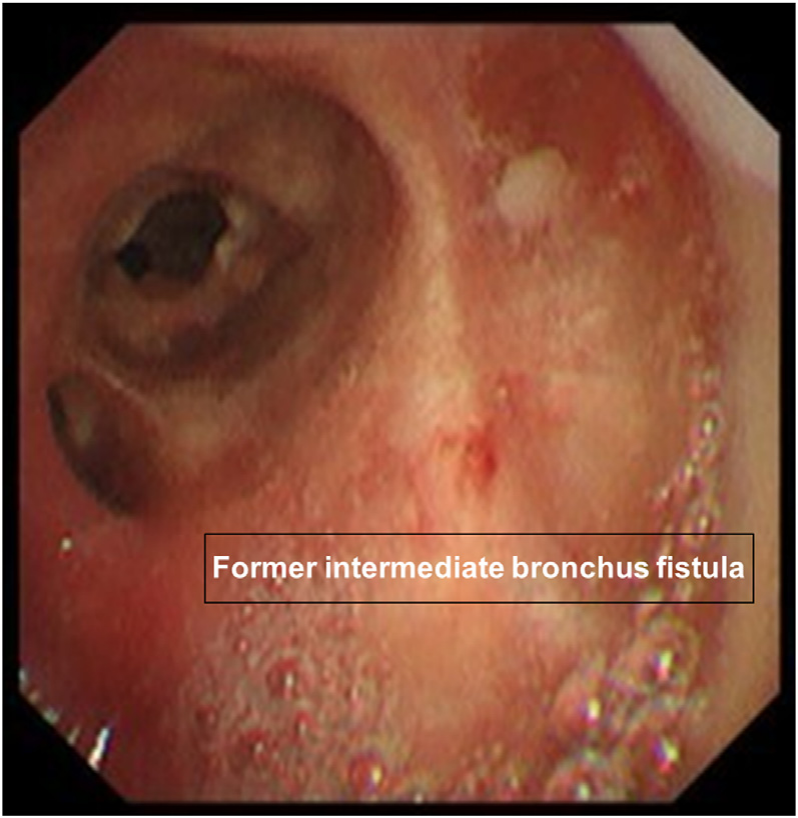
Bronchoscopy shows the former intermediate bronchus fistula.

## Comment

A postoperative bronchopleural fistula is a challenging condition that often proves difficult to treat. Berg and associates^1^ reported that 0.19% of 1594 patients who underwent segmentectomy developed a bronchopleural fistula, which was significantly lower than the probability of developing a bronchopleural fistula after lobectomy (0.5%). Although this case was not a segmental bronchial stump fistula, a similar report of a defect in the intermediate bronchus caused by ischemia and thermal damage to the membranous part of the intermediate bronchus by soft coagulation was reported by Takagi and colleagues.^2^ In the present case, the fistula was attributed to thermal damage from the blade side of a vessel-sealing system during lymph node dissection. Treatment for postoperative bronchopleural fistulas involves bronchoplasty, muscle flaps, suturing of mediastinal fat tissue, or omental filling, provided that infection is well controlled and primary closure is feasible. In cases where bronchoplasty is not viable, peripheral lung resection is sometimes considered. When infection control is not possible, fenestration is selected. In the present case, infection was sufficiently controlled, and it was decided to perform a temporary fistula closure, but this was thought to most likely result in a middle and lower lobectomy, which would cause significant loss of respiratory function. Therefore, to avoid middle and lower lobectomy, Dumon stent placement was deemed necessary to maintain bronchial patency. Dumon stents were originally placed under rigid bronchoscopy to relieve airway stenosis, but in recent years there have been scattered reports of Dumon stents being placed to close bronchopleural fistulas.^3^ Most of them were placed under rigid bronchoscopy, although 1 of them was placed into a bronchopleural fistula through fenestration.^4^ However, we could not identify any reports of insertion from the surgical field. In this case, the fistula was large, and it was difficult to place the stent under rigid bronchoscopy to fit the middle and basal bronchus inlets. Therefore, a Dumon stent was inserted directly into the fistula from the surgical field and replaced with autologous tissue. If this technique is used, we believe that using a stent that is slightly larger than the bronchial patency will prevent stent migration. This technique can be an effective treatment option for select cases of bronchopleural fistula in which insertion of a Dumon stent under rigid bronchoscopy or bronchoplasty is challenging, as highlighted in the present case.

For an intermediate bronchus fistula for which insertion of a Dumon stent under rigid bronchoscopy or bronchoplasty was difficult, direct insertion of a Dumon stent from the surgical field was effective in maintaining bronchial patency and preserving the peripheral lung.
